# Response to COVID-19 pandemic in the UAE: A public health perspective

**DOI:** 10.7189/jogh.11.03050

**Published:** 2021-03-27

**Authors:** Farida Al Hosany, Subhashini Ganesan, Shammah Al Memari, Shereena Al Mazrouei, Faheem Ahamed, Ashish Koshy, Walid Zaher

**Affiliations:** 1 Abu Dhabi Public Health Center (ADPHC), Abu Dhabi, UAE; 2 G42 Healthcare, Abu Dhabi, UAE

In the current pandemic of COVID-19 affecting countries across the globe, there are wide variations on how each country is handling the crisis. World leadership, economic status of the country, health care infrastructure, established public health foundations and various other factors play a role in it. Hence it becomes imperative to understand how the current pandemic, the biggest public health crisis, was handled in the UAE from a public health perspective.

## THE UAE IN COMBATING COVID-19

The World Health Organization (WHO) has released recommendations on strategic preparedness and handling of the COVID-19 pandemic [1]. Based on this, the article aims at discussing the UAE’s efficient combat of COVID-19 crisis under six priority areas of work.

### 1. Emergency response mechanism

The emergency response system of the UAE is managed by National Crisis and Emergency Management Authority (NCEMA) and the UAE government was vigilant in issuing the first alert of the new coronavirus outbreak, even before it was declared by WHO as a public health emergency of international concern [2].

The UAE has a comprehensive, government-funded health service as well as private health facilities which delivers a high standard of health care to the population.

During this pandemic, the entire health care system was prepared and alerted with protocols in place for efficient handling of the crisis

**Table 1** gives details of various circulars released by the government (DOH, ADPHC) related to the preparedness and management of the COVID-19 pandemic [3].

**Table 1:** Circulars and protocols released by the UAE government in handling COVID-19 [3]

Date	Title	Reference
21/1/2020	Novel Coronavirus alert	ADPHC-DG/C/01/2020
6/2/2020	Coverage of novel corona screening	USO/04/2020
2/3/2020	Novel Coronavirus circular (COVID-19)	ADPHC-DG/C/05/2020
3/3/2020	Novel Coronavirus circular (COVID-19)	ADPHC-DG/C/06/2020
8/3/2020	Coverage of corona virus disease 2019 (COVID-19) screening and treatment	USO/07/2020
22/3/2020	Diagnostic test for Corona (COVID-19)	USO/09/2020
24/3/2020	Diagnostic test for Corona (COVID-19)	USO/11/2020
24/3/2020	Licensed Healthcare Professional Rotation between Abu Dhabi Licensed Healthcare Facilities	USO/12/2020
26/3/2020	Effective hand sanitising products for COVID-19 pandemic	HLME/08/2020
26/3/2020	Safe working practices for OP pharmacies due to Covid-19 pandemic	HLME/07/2020
30/3/2020	Novel Coronavirus circular (COVID-19)	USO/16/2020
6/4/2020	Epidemiological Surveillance & Reporting of COVID-19 Cases	USO/18/2020
09/4/2020	Implementing medical home visits for corona virus (COVID-19) high risk categories	USO-22-2020
90/4/2020	Novel Coronavirus circular (COVID-19	USO-21-2020
06/5/2020	Coverage of corona virus disease 2019 (COVID-19) diagnostic and treatment services	USO-38-2020
24/5/2020	Covering corona virus (COVID-19) test in the activity based funded mandates (ABMs)	USO-51-2020
28/5/2020	Rational use of Personal Protective Equipment (PPE) as per Clinical Setting in Context of COVID-19 Response	USO/54/2020
28/5/2020	Testing of COVID-19 for Healthcare Workers	USO/52/2020
2/7/2020	Interim rules and requirements to minimize chance for exposures and to prevent spreading COVID-19 between HCWs.	USO/55/2020
7/72020	Updated National Protocol for Clinical Management and Treatment of COVID-19.	USO/57/2020
5/7/2020	Guidelines for COVID-19 Serology	USO/61/2020
12/7/2020	Coverage of Coronavirus disease 2019 (COVID-19) Diagnostic and treatment services	USO/68/2020
16/8/2020	Comprehensive COVID-19 Guideline for Healthcare Professionals in Abu Dhabi.	USO/80/2020
5/10/2020	COVID-19 screening test for all health care workers (HCWs)	USO/109/2020

HCW – health care worker

### 2. Risk communication and public engagement

Risk communication is a key factor during a pandemic and the UAE government executed a detailed awareness campaign that targeted different groups of the community and provided a daily update about the status of the disease locally and internationally. UAE adopted the mandatory use of face masks in March 2020 well ahead of the WHO recommending this policy [4]. Smart technology platform was effectively used in the UAE to combat COVID-19, for example, a chatbot service was established by MoHAP called “Virtual doctor for COVID-19. The “Doctor for every citizen” app was also made available for people for providing COVID-19 related information and services. The “Weqaya” platform, by NCEMA, was utilized to enhance awareness among the public on the ongoing COVID-19 public health crisis.”

The ALHOSN UAE app is the official integrated digital platform for COVID-19 tests in the UAE through which individuals can receive COVID-19 test results on their smartphones. Similarly, the StayHome app supported quarantine and isolation of patients [5].

The coronavirus helpline was established by the Ministry of Health and Prevention (MoHAP), as well as a dedicated hotline for mental health counselling to respond to psychological concerns and anxiety of people during the pandemic [5].

### 3. Case finding, contact tracing and management

The government of the UAE made remarkable progress in testing for the SARS-CoV-2 virus and in the management of cases. Algorithms for segregation, isolation, and treatment were released to guide health care professionals in handling COVID-19 cases (**Table 1**).

In the UAE, testing services were promptly provided in most health care facilities across the nation and for the ease of testing, drive through testing centres were established by the Department of Health – Abu Dhabi and SEHA. These centres were equipped with the most advanced testing systems, techniques, and globally accredited health care teams [6].

By the directives of His Highness Sheik Mohamed Bin Zayed Al Nahyan, Crown Prince of Abu Dhabi and Deputy Supreme Commander of the Armed Forces, free tests were provided to Emiratis, domestic workers in Emirati households, people of determination, pregnant women, residents aged over 50 years, people with chronic diseases, those with coronavirus symptoms and all contacts of the coronavirus patients.

Mass testing campaigns were conducted in crowded pockets where there was high risk of transmission [7], these intense measures were very effective in breaking the chain of transmission.

Field hospitals were set up in the UAE to ease the pressure on city-based hospitals and to aid faster response to COVID-19. The first field hospital in the UAE was opened at the Dubai World Trade Centre with a capacity of 300 beds, that can be expanded up to 3000 beds.

The Abu Dhabi Health Services Co (SEHA) has set up similar field hospitals at various locations and apart from these, a quarantine facility called Complex 3, with a capacity to quarantine 10000 patients was set up. The government vigorously worked to establish this in only 9 days [8].

All these facilities followed rigid COVID -19 protocols in handling and management of cases.

### 4. Surveillance of COVID-19

Circulars were released for all health care professionals and a series of training workshops were conducted to educate health care professionals about the disease diagnosis, reporting, and management to strengthen epidemiological surveillance and enhancing prompt reporting of all suspected COVID-19 cases to Abu Dhabi Public Health Centre (ADPHC). In addition, active surveillance was established to identify Severe Acute Respiratory Infections (SARI) admitted to different hospitals there are required to be tested for COVID-19 (**Table 1**).

**Figure:**
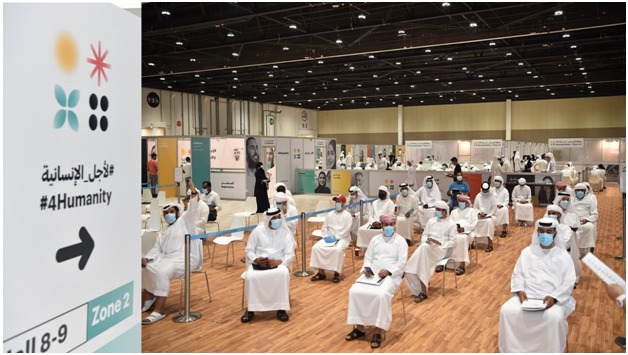
Photo: #4 Humanity Phase III trial, a key initiative to foster population health and to enhance UAE’s medical and research capabilities (#4Humanity Media Office, G42 Healthcare).

### 5. Vigorous public health measures to contain and combat COVID-19

The UAE is adopting the most demanding standard of two metres, double the distance recommended by the World Health Organisation. This had played a significant role in curbing the infections.

The UAE was one of the first countries to efficiently adapt remote learning in all private and government schools. The federal and local governments in the UAE adopted a remote working system to ensure the health and safety of its employees and customers. The government also issued orders to temporarily stop the distribution of all print newspapers, magazines, and marketing material to avoid transmission of virus by contact [9].

The UAE Government suspended all close gatherings and sports activities, commercial centers, shopping malls along with fish, meat, and vegetables markets were closed except for essential commodities. To contain the virus and to restrict entry of people into the UAE, which is an essential precautionary measure to curb the spread, the UAE suspended visas to all foreigners and all inbound and outbound passenger flights were cancelled [9]. The government announced enforcement of penalties to violators who threaten public health.

The country launched the world’s first phase III clinical trials (#4 Humanity clinical trial) for COVID-19 inactivated vaccine in July 2020 by G42 Healthcare in collaboration with Sinopharm CNBG, Department of Health, Abu Dhabi and UAE Ministry of Health and Prevention. This is a vivid example of national initiatives to foster population health and to enhance the UAE's medical research and development capabilities [10]. The vaccine is authorized for use and is given free of cost for all residents of the UAE. The UAE leads in top rankings for highest rate of vaccination [11].

SEHA plays a vital role in getting the residents vaccinated and it has vaccination centers established in various other Emirates for effective functioning and reach.

### 6. Societal response

The final priority work area for preparedness in dealing with this pandemic would be to develop all-of-society and business continuity plans. The UAE government is already leading the way in restarting business and announced a two-phase recovery plan to rebuild the economy [12]. The two biggest economies in the UAE, Dubai and Abu Dhabi have taken a holistic approach to economic revival. They have made decisions to relieve troubled businesses facing administrative penalties along with the stimulus package of US$79 billion for private sector in the first phase and in the next phase there is a long-term stimulus plan to speed up the recovery and encourage investments in digital economy like 5G networks and Artificial Intelligence, these are examples of how governments can implement a holistic approach to reboot economic growth. The UAE government along with the supreme Council of National security and NCEMA have released guidelines for Business continuity for UAE organizations.

## CURRENT STATUS OF COVID-19 PANDEMIC IN THE UAE

**Figure 1:**
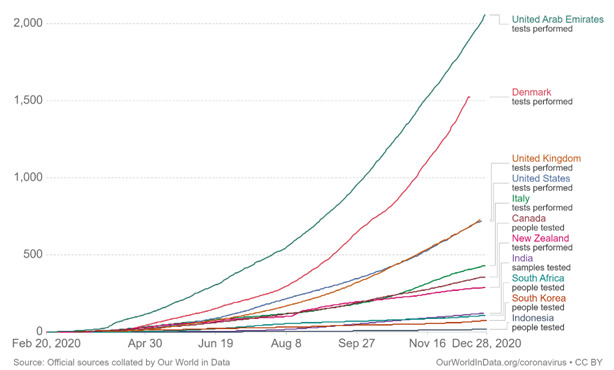
The graph shows the number of tests conducted per 1000 people.

The United Arab Emirates, as of 27 December 2020, has a COVID- 19 cases recovery rate of almost 89% and has recorded 669 COVID-19 deaths since the epidemic began [13]. The case fatality percentage is 0.3% and is lower compared to other developed countries. As of 29 December 2020, 209 734.9 tests per 100K population are done with the positive rate of 1.2% [14] (**Figure 1**). The UAE leads in global COVID-19 testing, the number of tests conducted per 1000 people is highest in the UAE compared to other developed nations.

## WAY FORWARD

The UAE moves forward with successfully reopening the economy and addressing the newer challenges of the pandemic like the newer variants of the virus.

The UAE government encourages research and development of newer technologies with regards to COVID-19 pandemic and aligned to the nation's objectives, G42 Healthcare introduced the LamPORE technology with strategic global partnerships that is now integral to the UAE national testing program [15].

G42 Healthcare has implemented a COVID-19 virus genomic sequencing study aimed at monitoring the pattern of spread and evolution of the virus since the start of the pandemic. This sequencing will also identify the emergence of new strains. Studies have already been published from the UAE on genomic sequencing of COVID-19 virus which has contributed to the understanding of the global transmission network of SARS-CoV-2 [16].

Thus, the UAE has been efficient and effective in handling the crisis of COVID-19. It continues to pioneer in health care and serves as a leading example in the Middle East in comprehensively protecting the population and responsibly reopening the economy.
